# Studying zebrafish nervous system structure and function in health and disease with electron microscopy

**DOI:** 10.1111/dgd.12890

**Published:** 2023-09-29

**Authors:** Sebastian M. Markert

**Affiliations:** ^1^ European Molecular Biology Laboratory Schwab Team Heidelberg Germany

**Keywords:** disease model, electron microscopy, nervous system, ultrastructure, zebrafish

## Abstract

Zebrafish (*Danio rerio*) is a well‐established model for studying the nervous system. Findings in zebrafish often inform studies on human diseases of the nervous system and provide crucial insight into disease mechanisms. The functions of the nervous system often rely on communication between neurons. Signal transduction is achieved via release of signaling molecules in the form of neuropeptides or neurotransmitters at synapses. Snapshots of membrane dynamics of these processes are imaged by electron microscopy. Electron microscopy can reveal ultrastructure and thus synaptic processes. This is crucial both for mapping synaptic connections and for investigating synaptic functions. In addition, via volumetric electron microscopy, the overall architecture of the nervous system becomes accessible, where structure can inform function. Electron microscopy is thus of particular value for studying the nervous system. However, today a plethora of electron microscopy techniques and protocols exist. Which technique is most suitable highly depends on the research question and scope as well as on the type of tissue that is examined. This review gives an overview of the electron microcopy techniques used on the zebrafish nervous system. It aims to give researchers a guide on which techniques are suitable for their specific questions and capabilities as well as an overview of the capabilities of electron microscopy in neurobiological research in the zebrafish model.

## Graphical abstract

To study nervous system function, electron microscopy techniques are crucial. However, many different techniques exist with different requirements and applications. This review is a resource to give a low‐bar introduction and overview of these techniques.

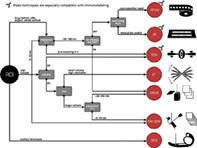

## 1 INTRODUCTION

Nervous systems are highly complex. This is true for all systems in animals, yet studying the nervous system poses some unique challenges. Here, signal transduction has to be precise, reliable, *and* fast. Whether the task is to report and encode signals from the environment, coordinating movement and heartbeat, or learning and cognition, the nervous system relies on highly evolved molecular machineries to carry it out. Zebrafish (*Danio rerio*) is an invaluable model for studying the nervous system, including with regard to human disease. Even though zebrafish are, of course, evolutionarily further removed from humans than mice, they also have some advantages compared to rodents as a model for biomedical research. They are transparent, which allows for analysis via light microscopy of intact animals. They also offer many mutants and transgenic reporter lines. However, to understand the functions and malfunctions of the nervous system, it is often not enough to study individually tagged molecules and proteins. The so‐called ultrastructure must be considered as well. Without observing the ultrastructure of the synapse, for example, we may never have figured out how quantal neurotransmitter release via synaptic vesicles works (Heuser et al., 1979). To understand these complex machineries, studying membrane dynamics and sub‐cellular morphology was crucial.

The ultrastructure is revealed by electron microscopy (EM). It is a powerful technique, but it can be challenging to wield. Since EM is performed in vacuum, it is not compatible with living material, which is full of liquid water that would boil off at negative pressures. Therefore, biological samples have to be prepared in certain ways to make them compatible with EM analysis. Depending on the tissue and cell type, as well as the questions asked, the choice of sample preparation and specific EM technique is crucial for a successful investigation.

In this review, applications of EM on the zebrafish nervous system are collected. Grouped by tissue, cell type, or disease model, the reader can find EM approaches that suit their questions or get an overview of ultrastructure analyses performed so far as well as the zebrafish models used in these works.

## 2 BRIEF OVERVIEW OF COMMON ELECTRON MICROSCOPY TECHNIQUES

EM is an old approach. The first electron micrograph was produced in 1931 by transmission EM (TEM) (Knoll & Ruska, 1932). Since then, many different techniques and applications have evolved. In classical TEM, images are produced when the biological material inside ultrathin plastic sections (~40–100 nm) casts shadows on photo plates, or nowadays on digital cameras. Electron‐dense structures like proteins scatter more beam electrons and thus cast a shadow. In contrast to that, scanning EM (SEM) uses a moving electron beam to scan surfaces while static detectors collect scattered electrons for any given pixel in the image. This can only be used superficially, at the surface of material, but by cutting into the sample with a diamond knife (serial block face SEM [SBEM]) (Denk & Horstmann, 2004; Hughes et al., 2014; Smith & Starborg, 2019) or a focused ion beam (FIB‐SEM) (Bushby et al., 2011; Heymann et al., 2009; Knott et al., 2011), the inside can be sequentially imaged as well, by alternating scanning and removal of material. This destroys the sample in the process, but large 3D volumes can be acquired this way without gaps.

EM reveals ultrastructure. While this is the selling point of EM, it can also be a downside. All proteins, lipids, nucleotides, etc., are imaged. Localizing a specific molecule of interest can be challenging here. This is easy with light microscopy (LM), however (Costantini et al., 2015; Hibbs, 2004; Tsien, 1998). Fluorescent tags can be used to label practically any molecule of interest, but the ultrastructural context is lost. To get the best of both worlds, correlative light and electron microscopy (CLEM) can be employed (de Boer et al., 2015; Müller‐Reichert & Verkade, 2012). This means that EM and LM are combined to put molecular identity in its full ultrastructural context. Because EM and LM are generally incompatible, however, this can be tricky. EM generally relies on dehydration and strong fixation and heavy metal staining, whereas LM works best in lightly fixed, hydrated samples. Nevertheless, many CLEM protocols have emerged with clever compromises to make them work in both imaging modalities (e.g., Karreman et al., 2016; Kukulski et al., 2011; Markert et al., 2016; Micheva & Smith, 2007).

It is also possible and has been used for many decades to mark molecules of interest with gold particles, for example via gold‐conjugated antibodies (Faulk & Taylor, 1971; Flechsler et al., 2020; Slot & Geuze, 1985). This way, the gold particles reveal the location of these molecules in the electron micrograph. The downsides of this technique include the low labeling density compared to fluorophore‐coupled markers and the difficulty of finding the tiny gold particles in the sample (Fabig et al., 2012; Schwarz & Humbel, 2014).

Genetic labeling can also be used in EM. The two main approaches are miniSOG (Shu et al., 2011) and APEX (Lam et al., 2015; Martell et al., 2012). MiniSOG (mini Singlet Oxygen Generator) is a protein tag that locally creates polymers that attract osmium tetroxide (a common heavy metal stain in EM). This way, osmium accumulates in the immediate surroundings of the miniSOG and an electron‐dense label is produced. However, miniSOGs are dependent on light activation to work, which may pose a challenge for large or opaque samples. APEX (“ascorbate peroxidase enhanced”) (Martell et al., 2012) and its improved version APEX2 (Lam et al., 2015) also create osmophilic polymers, but are independent of light. Both miniSOG and APEX are genetically encoded and thus can in principle be used to label any cell or protein of interest, similarly to how fluorescent protein tags are used in light microscopy. However, multiple labeling is not possible with these techniques. While miniSOG or APEX have not been used in zebrafish neurobiology to date, they seem promising techniques, especially considering the vast repertoire of genetic zebrafish lines available.

The key to the art of EM lies within sample preparation. How a sample is fixed, stained, and embedded is vastly more important than the choice of microscope or camera. In “classical” EM, samples are chemically fixed with formaldehyde and/or glutaraldehyde and then dehydrated in an ethanol series of increasing concentrations. Next, the ethanol is replaced by a volatile organic solvent (typically propylene oxide or acetone) and finally the solvent is replaced by resin diluted in the same solvent, with increasing resin concentrations, before the sample is embedded in pure resin and cured (hardened by polymerization of the resin monomers). Nowadays, sample preparation by high‐pressure freezing and freeze substitution is also used a lot (Helmprobst et al., 2015; Nixon et al., 2009). Here, samples are immobilized rapidly by freezing under 2000 bars of pressure. Within milliseconds, the water in the sample enters a vitrified state. That means, it assumes an amorphous, glass‐like state instead of forming crystal structures that would destroy the ultrastructure of the sample. The vitrified ice is then replaced by acetone and the sample is fixed and/or stained before it is brought back up in temperature for resin embedding and curing. While being more time consuming, costly, and labor‐intensive, high‐pressure freezing and freeze substitution offer the advantage of better structure preservation in many cases. The typical shrinking and wrinkling and other artifacts of classical chemical fixation are much reduced or eliminated in many cases (Rostaing et al., 2004; Royer & Kinnamon, 1996). Also, snapshots of fast dynamic cellular processes can be caught in action, since cryo‐immobilization is much faster than chemical fixation (milliseconds versus seconds to minutes). In general, there is a large variety of approaches and protocols for sample preparation. Often, these are tissue‐ or cell type‐specific, or they are optimized to visualize different aspects of the ultrastructure, for example, focusing on highlighting membranes versus cytoplasmic proteins. Table 1 gives an overview of common sample preparation reagents and compounds.

**TABLE 1:** Introduction and overview of some of the common reagents and compounds used for sample preparation for electron microscopy.

Fixation			
Reagents	Notes	Pros	Cons
Formaldehyde	Wide‐meshed crosslinking, can be combined with glutaraldehyde	+ good preservation of antigenicity + often compatible with immunostaining	− preservation of ultrastructure can be compromised
Glutaraldehyde	Tight‐meshed crosslinking, can be combined with formaldehyde	+ excellent preservation of ultrastructure	− often kills antigenicity − usually not compatible with subsequent immunostaining
Osmium tetroxide	Acts both as fixative and as staining agent	+ excellent preservation of ultrastructure + lipophilic, excellent staining of membranes	− highly toxic and volatile − proteolytic if left on for too long − usually not compatible with subsequent immunostaining

Staining			
Metal stains	Notes	Pros	Cons
Uranyl acetate	Universal EM staining agent gives contrast to proteins, phospholipids, and nucleic acids and also has fixating properties	+ readily available in any EM facility + quick and straightforward staining procedure + intensity of stain can easily be influenced by choice of solvent (water, ethanol, methanol)	− radioactive and toxic − increasingly hard to buy due to safety concerns − not a good stain for membranes
Lead citrate	Binds to osmium and uranyl acetate and thus enhances contrast	+ readily available in any EM facility + cheap	− easily reacts with CO_2_ from the air and creates unwanted precipitates
Osmium tetroxide	Acts both as fixative and as staining agent lipophilic	+ excellent stain for membranes + also stains proteins and nucleic acids	− highly toxic and volatile − not viable as on‐section stain, only pre‐embedding
Potassium permanganate	Mostly used as membrane stain	+ cheap + less toxic than other staining agents + can yield excellent membrane contrast	− can be highly extractive for cytoplasm − can create artifacts − not a good stain for cytoplasm

Embedding			
Resins	Notes	Pros	Cons
Epon	Epoxy resin, similar to araldite	+ creates covalent bonds with the sample and thus stabilizes it + easy to section	− less suitable for immunostaining − generally only usable at room temperature
Araldite	Epoxy resin, similar to epon	+ creates covalent bonds with the sample and thus stabilizes it + easy to section	− less suitable for immunostaining − generally only usable at room temperature
Durcupan	Epoxy resin, harder than epon or araldite, good choice for FIB‐SEM	+ creates covalent bonds with the sample and thus stabilizes it + relatively easy to section + more stable in FIB‐SEM applications	− more viscous, harder infiltration − generally only usable at room temperature
LR White	Acrylic resin	+ more hydrophilic than epoxy resins + better compatibility with immunostaining + can be used at lower temperatures (0°C and up) + can be polymerized with heat or UV light	− no covalent bonds with the sample, less stable sections − brittle, harder to section − only polymerizes under exclusion of oxygen
Lowicryl HM20	Acrylic resin	+ more hydrophilic than epoxy resins + better compatibility with immunostaining + can be used at very low temperatures (−40°C and up) + can be polymerized with UV light	− no covalent bonds with the sample, less stable sections − brittle, harder to section

All EM techniques mentioned so far are generally performed on samples that have been dehydrated, stained with heavy metals, and embedded in plastic resins. These steps can be avoided with cryo‐EM (Adrian et al., 1984; Dubochet et al., 1988; Milne et al., 2013). Here, the sample is cryo‐preserved and sectioned (if necessary) and imaged in the frozen state. This method is arguably more native because the sample did not undergo chemical alterations and additions. However, since the sample does not contain heavy metals, contrast is extremely low and the frozen sections are easily destroyed by the electron beam, requiring imaging at low electron doses. Thanks to technical advances, cryo‐EM can offer impressive images nowadays, though (Danev & Baumeister, 2016; Mahamid et al., 2016; Paul et al., 2020). Most applications are in single‐particle studies, especially for protein structures (Fernandez‐Leiro & Scheres, 2016), but tissue can be imaged as well (Al‐Amoudi et al., 2004; Klumpe et al., 2022).

**FIGURE 1:**
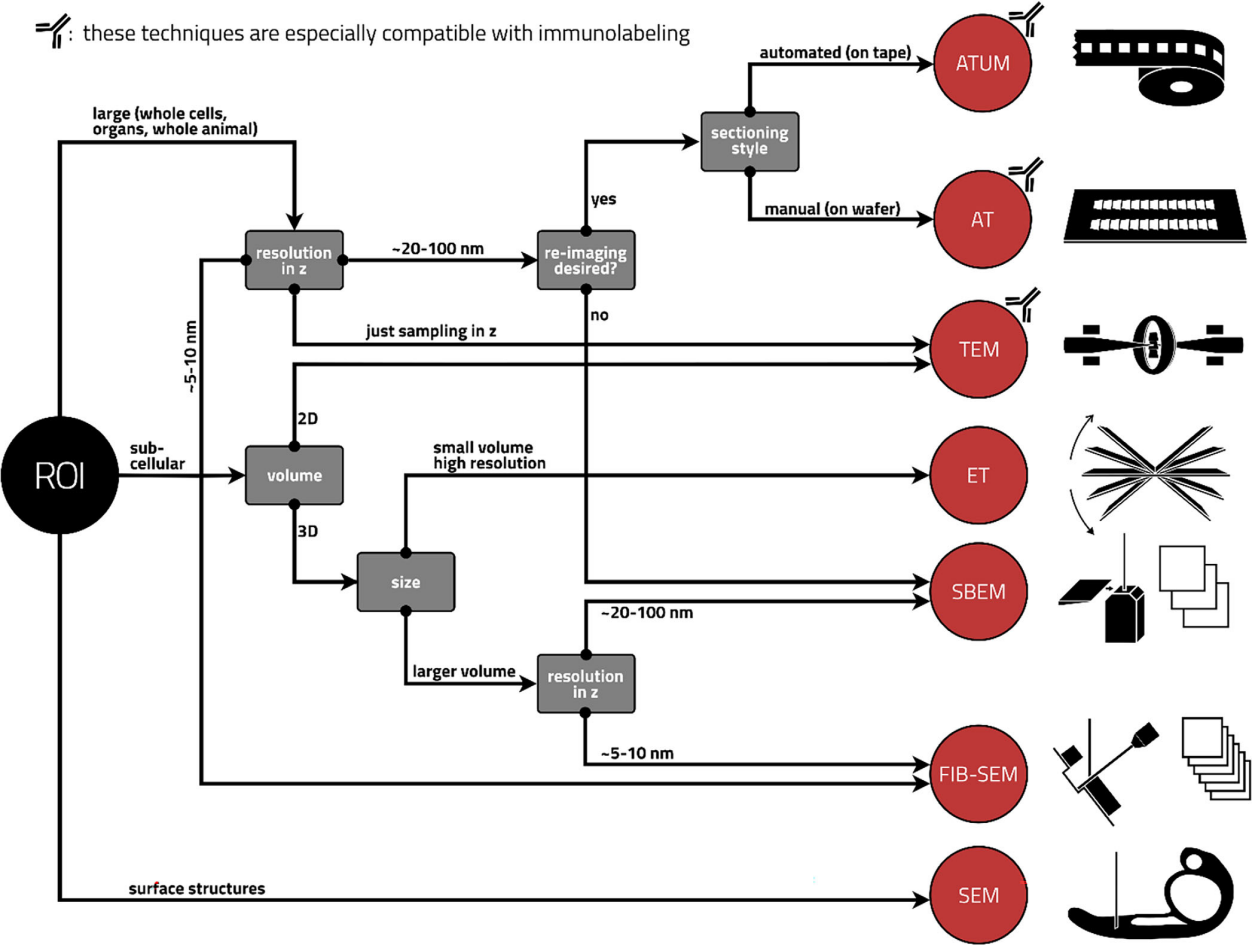
Decision tree for choosing the appropriate electron microscopy technique (red circles) for a given region of interest (ROI). Pictograms depict the different techniques. AT, array tomography; ATUM, automatic tape‐collecting ultramicrotome; ET, electron tomography; FIB‐SEM, focused ion beam scanning electron microscopy; SBEM, serial block face scanning electron microscopy; SEM, scanning electron microscopy; TEM, transmission electron microscopy.

All these techniques have been employed to study structure and function in the zebrafish nervous system. Zebrafish has emerged as a powerful model for many human diseases of the nervous system. For an overview of common EM techniques and their pros and cons, see Table 2. For a decision tree to choose an EM technique appropriate for a particular region of interest and question, see Figure 1.

**TABLE 2:** Overview of common electron microscopy techniques with their pros and cons.

Technique	Full name	Pros	Cons
TEM	Transmission electron microscopy	+ “standard” EM, very accessible + wide range of magnifications possible + samples can be re‐examined (as long as grids remain intact) + compatible with practically all sample types, from whole brains to single vesicles (but no single particle)	− resolution in *z*‐axis limited by section thickness − less suitable for 3D volumes − sectioning (ultramicrotomy) requires skill
SEM	Scanning electron microscopy	+ relatively cheap and accessible + also suitable for large samples (including whole adult zebrafish) + best technique for 3D surfaces + extremely wide range of magnifications + re‐examination possible	− only suitable for surfaces (but also works as TEM substitute on sections) − prone to artifacts from dehydration (shrinking, wrinkling) − re‐examination can be limited by contamination of the sample
ET	Electron tomography	+ good accessibility + highest resolution in *x*, *y*, and *z*‐axes + high‐resolution 3D information	− takes time to acquire − processing necessary to calculate tomograms − only suitable for small volumes, about a mikron cubed at max
STEM	Scanning transmission electron microscopy	+ both transmitted and scattered electrons can be recorded + combination of TEM and SEM + suitable for thicker sections	− less accessible than TEM or SEM − contamination of the sample over time − lower resolution than TEM
AT	Array tomography	+ compatible with immunolabeling + compatible with correlative light and electron microscopy + re‐imaging of ultrastructure possible + sections are on wafer or glass slide and thus highly stable	− manual sectioning and placing section arrays on wafers requires skill − resolution in *z*‐axis limited by section thickness − resolution in *x*‐ and *y*‐axes limited compared to TEM
ATUM	Automatic tape‐collecting ultramicrotome	+ compatible with immunolabeling + compatible with correlative light and electron microscopy + re‐imaging of ultrastructure possible + automated sectioning + suitable for very large volumes + sections are on tape and thus highly stable	− high demands on sample and block quality for automatic sectioning − resolution in *z*‐axis limited by section thickness − resolution in *x*‐ and *y*‐axes limited compared to TEM
FIB‐SEM	Focused ion beam scanning electron microscopy	+ no sectioning, i.e., no compression and no folds + best technique for isotropic 3D imaging	− not very accessible − regions of interest can be hard to find − relatively small volumes (typically one cell) − resolution in *x*‐ and *y*‐axes limited − “curtaining” artifacts − destructive imaging, re‐imaging impossible
SBEM	Serial block face scanning electron microscopy	+ imaging of the block face, so no compression or folds + great technique for high volumes + isotropic resolution possible	− not very accessible − resolution in *x*,‐ *y*‐, and *z*‐axes limited − often problems with contrast and charging − high demand on sample preparation quality − destructive imaging, re‐imaging impossible
cryo‐EM	Cryogenic electron microscopy	+ imaging of native tissue, no fixation, dehydration, staining + very high resolution possible + suitable for the analysis of protein structures	− not very accessible, expensive − samples get destroyed with continued imaging − re‐imaging only at progressively deteriorating quality − low contrast due to lack of metal staining − demanding sample preparation

## 3 BRAIN AND SPINAL CORD

Much excellent work has been done to establish zebrafish as a model for the vertebrate nervous system, often with the help of EM. One crucial step was to differentiate neurons and glia in cell cultures, which are invaluable for in vitro studies. Ghosh and colleagues managed to culture neurons and astrocytes derived from early embryos (Ghosh et al., 1997). They used TEM to evaluate the morphology and ultrastructure of their cells. Neurons showed neurites with microtubules, synaptic vesicles, and growth cones. Thus, with one EM preparation they confirmed that the cells had seemingly successfully differentiated into neurons and astrocytes.

Not only embryonal cell culture is possible, but also explants from the adult zebrafish brain can survive several days in culture. Unlike land vertebrates, zebrafish can regenerate brain damage fully. It has been shown with TEM that the explants form neural tube‐like structures reminiscent of those seen in embryos during the development of the brain (Peguero et al., 2022). TEM also confirmed that the explants have healthy‐looking cellular ultrastructures. In the same study, SEM was used to observe the surface of the explants at different time points. Protrusions of regenerating tissue formed after only 4 days in culture. Without SEM, these structures would have been hard to observe in the translucent explant.

It is also possible to combine TEM and SEM to scanning transmission EM (STEM). In microscopes capable of STEM, transmitted as well as scattered electrons can be detected. Kuipers and colleagues published a detailed protocol on how to obtain large‐scale 2D STEM data sets in larvae on healthy and injured brain (Kuipers et al., 2016). To image brain damage, they used a non‐invasive ablation model and imaged cellular and subcellular changes.

Zebrafish is not only a model for brain injury, but also for neurotoxicity. One neurotoxic substance that is important for human health is the environmental pollutant methylmercury (MeHg), which can accumulate in the body by eating contaminated fish. So it seems only fitting to study the effects of MeHg on the brain in a fish model. Cambier and colleagues kept adult zebrafish in water contaminated with environmentally relevant amounts of MeHg and examined its influence on the brain (Cambier et al., 2012). Histological examination did not yield any significant differences between contaminated brains and controls. However, when they employed TEM, they found that the nuclei of the optic tectum were reduced in size in the MeHg treatment group. Affected brain areas also showed a reduced cell density. This shows that it can pay off to examine the ultrastructure if experimental effects are expected to be subtle.

In contrast to this, Lindsey and colleagues used TEM and also some SEM specifically to create a detailed and comprehensive description of the adult forebrain, especially with regard to adult neurogenesis (Lindsey et al., 2012). They describe the ultrastructure of all the different cell types and offer a classification scheme for the first time. In addition, they use the classical technique of immunolabeling with gold‐conjugated antibodies to confirm cell identities. Such comprehensive maps are of great value to the zebrafish community.

In a similar manner, in their foundational work, Kuwada and colleagues used TEM to describe the development of the spinal neurons and their tracts in the early zebrafish embryo (Kuwada et al., 1990).

While such TEM atlases are invaluable for understanding the structure of the nervous system, they are usually not suitable for reconstructing the connections, the synaptic circuitry. This requires complete 3D data sets without any gaps, so the neuronal projections can be followed and traced from soma to synapse to retain correct identification. Usually, the volumes required here are, in EM terms, gigantic. Thus, circuit reconstructions require specialized techniques and expertise. They are collected under their own heading, “Circuits and Connectomics” (see below). In the following, we will continue with more conventional EM studies of parts of the brain and spinal cord.

### 3.1 Myelin

Myelination of axons is a characteristic of jawed vertebrates (gnathostomes) and its loss is a feature of several neurodegenerative autoimmune diseases. Myelin sheaths are produced by certain glia cells called oligodendrocytes in the central nervous system and Schwann cells in the peripheral nervous system. Myelination greatly and crucially increases the rate of nerve signal transmission (Bunge, 1968). To establish zebrafish as a model for myelination, Brösamle and Halpern characterized it in zebrafish larvae (Brösamle & Halpern, 2002). In their foundational study, they described the ultrastructure of developing myelin with TEM and localized transcripts of proteins involved in myelination, laying the groundwork for dissecting this process with genetic approaches.

A few years later, Pogoda and colleagues published their genetic screen for mutations in myelin‐associated genes (Pogoda et al., 2006). They identified 13 mutations in 10 genes that disrupted myelination. These serve as models for human diseases involving myelin. TEM was a crucial part of their study to characterize the ultrastructural changes in myelination in the mutants.

Recently, Siems and colleagues published the proteome profile of myelin in the zebrafish brain (Siems et al., 2021), highlighting the similarities between myelin in fish and rodents, as well as the differences. They used TEM to confirm the presence of myelin sheaths in their purification fractions and they used immunogold labeling on cryosections to localize proteins.

### 3.2 Mauthner neuron

The so‐called Mauthner neuron has long been established as an important model neuron in fish and amphibians. The Mauthner neuron integrates several sensory inputs and initiates the escape response. Due to its large size and consistency in location and morphology, it is an excellent choice for electrophysiological analyses. This made it into a classical model for synaptic transmission. More than 40 years ago, Kimmel and colleagues published a beautiful and comprehensive study on the morphogenesis and synaptogenesis of the Mauthner neuron (Kimmel et al., 1981). They used serial light microscopy to reconstruct the entire neuron and also obtained electron micrographs from the same sample at about 2.5‐μm intervals with classical TEM. A heroic effort, reminiscent of modern CLEM and volume EM approaches.

Speaking of which, 39 years later Strobel and colleagues presented a modern CLEM protocol to study identified neurons and used the Mauthner neuron as their example (Strobel et al., 2020). They labeled the Mauthner neuron specifically by retrograde tracing with fluorescent rhodamine dextran. This label could be preserved in the sample preparation process of high‐pressure freezing and freeze substitution. This yielded near‐native structure preservation while allowing them to find and analyze the Mauthner neuron in arrays of serial plastic sections via the rhodamine fluorescence. This CLEM approach is an advancement of the array tomography technique (Markert et al., 2016, 2017; Micheva & Smith, 2007). For array tomography, long arrays of consecutive sections are placed on silicon wafers or glass slides and then the sections are imaged with fluorescence as well as scanning EM. Fluorescent labels are either created before embedding and preserved or added on top of the section, e.g., via immunolabeling.

Svara and colleagues published an impressive whole‐brain study which will be discussed below, but it should be mentioned here that their study included a comprehensive analysis of the connectivity of the Mauthner neuron (Svara et al., 2022).

### 3.3 Blood–brain barrier

The blood–brain barrier is highly complex and difficult to study in vivo. It is a semipermeable protective barrier formed by endothelial cells around blood vessels to regulate the influx of molecules into the central nervous system (Daneman & Prat, 2015). To help establish zebrafish as a model for blood–brain barrier research, Eliceiri and colleagues provide an overview of the techniques that can be used to study blood–brain barrier integrity in adult fish (Eliceiri et al., 2011). They use TEM for ultrastructural analyses of capillaries, the associated basal lamina, and astrocytes.

The blood–brain barrier is also pharmacologically important. Drugs that target the brain need to be able to pass through the barrier. One strategy to get drugs through is loading them into carriers like the cubosomes, lipid‐based nanocarriers. Azhari and colleagues recently showed for the first time that their cubosomes were able to get drugs across the blood–brain barrier in zebrafish (Azhari et al., 2021). They confirmed the arrival of gold‐labeled cubosomes in the brain via TEM. They also imaged the cubosomes themselves with cryo‐TEM to show their ultrastructure.

### 3.4 Synapses

In the nervous system, signals are passed between cells via so‐called synapses. Fundamentally, they can be divided into chemical and electrical synapses. Electrical synapses are formed by gap junctions which directly electrically couple cells by allowing ion flux through pores that span the plasma membranes of both cells (Evans & Martin, 2002; Goodenough & Paul, 2009; Nielsen et al., 2012). In chemical synapses, signaling molecules (“chemicals,” neurotransmitters or neuropeptides) are released by one cell and received by one or more others (Cioni et al., 2002; Gray, 1969; Peters & Palay, 1996; Zupanc, 1996). They can be further divided into asymmetrical (typically excitatory), symmetrical (typically inhibitory), and neuropeptide releasing types. Chemical synapses contain highly complex and intricate machinery to ensure faithful signal transduction between cells via neurotransmitter release from synaptic vesicles. Many proteins are involved in this machinery and when they are malfunctioning due to toxins or genetic mutations, the effects on the organism are often severe or even deadly. Thus, understanding synaptic function on the molecular level is an important goal of neurobiology. Since synaptic vesicles are well below the resolution limit of light microscopy, EM is often the only way to study synaptic processes and membrane dynamics in detail. Even then, classical TEM often does not offer enough resolution in the *z*‐axis to characterize vesicle pools and active zones of exocytosis in 3D. To mitigate this, a sample section can be tilted and imaged at multiple angles. From such tilt series, electron tomograms can be reconstructed. Such electron tomograms easily reach (virtual) *z*‐resolutions of 1 nm and are the technique of choice for sub‐synaptic analyses. Helmprobst and colleagues demonstrated this nicely in presynapses of the neuromuscular junction in zebrafish larvae (Helmprobst et al., 2015). They compared the presynaptic architecture of different ages (4 vs. 8 days post‐fertilization [dpf]) and fixation methods (chemical fixation vs. high‐pressure freezing/freeze substitution) and offered valuable quantitative data, making this study into a benchmark of synaptic architecture analysis in zebrafish.

In a follow‐up publication, Kaltdorf and colleagues (including two of the authors of the previous study) offered a workflow for automated registration and quantification of synaptic vesicles in electron tomograms (Kaltdorf et al., 2017). A subsequent update can even classify vesicles into clear core and dense core vesicles automatically, though they showcased this in synapses of *Caenorhabditis elegans* only, and not in zebrafish so far (Kaltdorf et al., 2018). But since their classification algorithm can be trained, it can be adapted to any synapse type and species, in principle. The author of this review has successfully employed their tools in a study on the effects of a protein implicated in amyotrophic lateral sclerosis on synaptic architecture in *C. elegans* (Markert et al., 2020).

In another exciting study, Bayés and colleagues looked at the evolution of the proteome in synapses of adult zebrafish (Bayés et al., 2017). They compared it to the synaptic proteasome in mice and thus offer insight into the effects of the whole‐genome duplication of teleost fish on synapse composition. They employed TEM to offer the most comprehensive ultrastructural description of the asymmetrical (typically excitatory) synapse types in the adult zebrafish brain to date.

### 3.5 Alzheimer's disease

Alzheimer's disease is a widespread neurodegenerative disease we are desperately trying to find treatments for. The hallmark of Alzheimer's pathology is the accumulation of amyloid‐β plaques in the brain. Bhattarai and colleagues introduced amyloid‐β into the brains of adult zebrafish and thus established an Alzheimer's disease model (Bhattarai et al., 2016). They found that amyloid‐β upregulated neural stem cell regeneration and neurogenesis, which is not the case in humans. The authors discuss how upregulating neurogenesis in patients could be achieved and if this could be a viable treatment. They used TEM to show that the amyloid‐β they injected into the fish does indeed form β‐sheet plaques, a crucial control for establishing zebrafish as an Alzheimer's disease model.

The amyloid precursor protein (APP) has a crucial role in the pathology of Alzheimer's disease, but its functions in the brain are still not clear. Song and Pimplikar knocked down APP in a zebrafish model and characterized the resulting phenotype with TEM and other methods (Song & Pimplikar, 2012). They found a defect in motor axon outgrowth that could be rescued by human APP, but not by mutated APP implicated in familial Alzheimer's.

Even though zebrafish can evidently be a valuable model for Alzheimer's research, one should always stay aware of the limits of any model. This was showcased by Newman and colleagues in their TEM study of zebrafish larvae (Newman et al., 2017). They used morpholinos to knock down proteins that are mutated in patients suffering from familial Alzheimer's disease. In mice, knockout of these genes increased the length of mitochondrion–endoplasmic reticulum appositions. Newman and colleagues measured the lengths of these appositions in TEM images of the spinal cord precursors of their zebrafish embryos injected with morpholinos. They obtained and published an essentially negative result. They were not able to find any differences in mitochondrion–endoplasmic reticulum appositions in fish.

In one of the most exciting recent studies, Javed and colleagues inhibited the toxicity of amyloid‐β in zebrafish. The plaques vanished and the fish recovered mobility and cognitive function, achieving essentially the equivalent of a successful treatment of Alzheimer's disease (Javed et al., 2019). They managed this by treating the fish with casein‐coated gold nanoparticles that get across the blood–brain barrier and sequester amyloid‐β. TEM was essential for confirming the presence of their gold nanoparticles in the brain. They were also able to directly show the sequestration of amyloid‐β by the nanoparticles with the negative staining technique (Harris, 2007) and TEM.

One of the environmental risk factors for Alzheimer's disease is aluminum. Chen and colleagues have shown that aluminum oxide nanoparticles accumulate in zebrafish embryos and cause progressive impairment of learning and memory in adulthood (Chen et al., 2020). With TEM they found damaged mitochondria and increased autophagy in the telencephalon. These phenotypes are reminiscent of Alzheimer's pathology. Gao and colleagues therefore sought to establish an Alzheimer's disease model by exposing zebrafish to different concentrations of aluminum (Gao et al., 2022). The fish developed memory and learning impairments and expression of proteins related to Alzheimer's increased. When they specifically inhibited necroptosis, which has been shown to improve symptoms in other zebrafish models, their fish recovered. They further used TEM to show how necroptosis inhibition improved the ultrastructure in neurons of the telencephalon, though they did not quantify these effects. These results offer evidence that their zebrafish model could be valuable for studying aluminum‐related Alzheimer's disease.

### 3.6 Parkinson's disease

Zebrafish can also serve as a model for Parkinson's disease. In early onset Parkinson's, the most commonly mutated gene is *parkin*. Flinn and colleagues developed a zebrafish model deficient in Parkin that can be used for fast screening of disease‐modifying compounds (Flinn et al., 2009). In their study, they describe the effects of Parkin depletion on dopaminergic neurons and also show ultrastructural alterations in the muscles via TEM.

All these Alzheimer's and Parkinson's studies show how zebrafish can offer a serious alternative to mouse models for neurodegenerative diseases, at high throughput and low cost. They also show the value of EM techniques for disease studies.

### 3.7 Infection

How zebrafish can also serve as a model for infections of the nervous system has recently been shown by Wang and colleagues (Wang et al., 2021, 2022). The authors ring the alarm bell about covert mortality nodavirus (CMNV). They show that this marine shrimp virus can also infect zebrafish, a freshwater fish. CMNV could be localized in different tissues via TEM, including eye and mesencephalon, where it causes lesions that lead to increased mortality and altered behavior.

## 4 PERIPHERAL NERVOUS SYSTEM

The gastrointestinal tract possesses its own nervous system. This enteric nervous system (ENS) is responsible for many essential gut functions, like peristalsis, homeostasis of the intestinal barrier, and secretion of hormones. Zebrafish has emerged as a valuable model for studying ENS development. Recently, Baker and colleagues published a detailed immunohistochemical and ultrastructural analysis of the ENS in larvae at 6 dpf, which seems to be a crucial time point for ENS patterning (Baker et al., 2019). This work thus lays the groundwork for future functional and biomedical studies.

Another poorly understood part of the peripheral nervous system are the so‐called paraneurons located in epithelia. For example, the epidermis of the caudal fin in adult zebrafish contains superficial serotonergic neurons of unknown function. König and colleagues examined these paraneurons during fin regeneration and found that they are restored during fin regeneration but are not responsible for the regeneration itself (König et al., 2019). They hypothesize that they play a role in hydrodynamic sensing, since these cells seemed to preferentially grow in areas with more turbulent fluid dynamics. TEM and SEM were employed in this study to analyze the ultrastructure of paraneurons and surface features of the epidermis. However, they only infer the identity of the cells they imaged via TEM. Correlative approaches like array tomography (Markert et al., 2017; Micheva & Smith, 2007) could have helped here to identify the cell types with confidence.

## 5 HAIR CELLS

Hair cells are mechanosensory neurons and transduce vibrations of air or water. They occur in the vestibular and auditory systems of all vertebrates and in the lateral line organ of fish. Because the hair cells of the lateral line organ are exposed on the surface of the body and thus easily accessible, zebrafish can be a convenient model for studying hair cells. Harris and colleagues used SEM to describe the surface morphology of these hair cells in great detail (Harris et al., 2003). They analyzed the effects of increasing concentrations of neomycin on their appearance and survival. Hair cells are extremely sensitive to neomycin. Then, they analyzed the recovery of the hair cells and found that they could regenerate within 24–48 h after treatment. With this work, they offer a model and benchmark for studying hair cell survival and regeneration in vertebrates which potentially could also function as a model for hearing loss and recovery.

Hair cells have a special kind of synapse, the so‐called ribbon synapse. In these synapses, synaptic vesicles are lined up at proteinaceous ribbons. Presumably, this helps with delivering the vesicles to the active zone for exocytosis, though many mechanisms of ribbon synapse function remain poorly understood. Obholzer and colleagues investigated the ribbon synapses of *vglut3* mutant zebrafish (Obholzer et al., 2008). The gene *vglut3* encodes a vesicular glutamate transporter and is expressed in hair cells of the ear and lateral line organ exclusively. The mutant fish lack vestibulo‐ocular and acoustic startle reflexes. TEM of the ribbon synapses revealed that the number of vesicles attached to the ribbons is significantly reduced, pointing to a defect in synaptic transmission. The authors hypothesize that Vglut3 is involved in vesicle biogenesis and/or tethering to the ribbon. Further progress in understanding the molecular mechanisms of ribbon synapse function will crucially depend on EM techniques.

Dow and colleagues investigated the wiring of the hair cells in the neuromasts of the lateral line (Dow et al., 2018). They used SBEM to acquire whole neuromasts of 2–4 dpf larvae and were able to reconstruct their circuitry. They thus gained insight into the characteristics and principles of neuromast wiring.

In a very recent study, Riley and colleagues deployed SBEM to obtain the 3D ultrastructure of hair cells of the inner ear for the first time (Riley et al., 2023). They compared 5 dpf wild‐type hair cells to those of a zebrafish model for Usher syndrome type 1B, which causes severe deafness and progressive loss of vision in humans. Surprisingly, while the hair cell synapses were a bit smaller in the disease model, the overall synaptic architecture was essentially unchanged. The authors hypothesize that the defects are caused more downstream, for example by disruption of the circuitry. Also, since segmentation and data analysis of huge SBEM volumes is very labor‐intensive, this study did not yet investigate other interesting aspects, like the number of tethered vesicles or potential changes in mitochondrial composition. Without a doubt, their volume EM data set will be invaluable for future analyses.

Even though SBEM can acquire huge volumes, the resolution in the *z*‐axis is limited by section thickness, with typical values of 20–50 nm. For better *z*‐resolution, FIB‐SEM can be used. It is not uncommon to achieve isotropic resolutions of 5 nm in the *x*‐, *y*‐, and *z*‐axes. However, acquisition volume is more limited. Schieber and colleagues showcased the power and application of FIB‐SEM in a book chapter, where they targeted and imaged a neuromast in a zebrafish larva (Schieber et al., 2017). By embedding the larva in a minimal amount of resin, they were able to select the region of interest precisely and image it directly, without the need for trimming the block.

## 6 VISUAL SYSTEM

Many of the countless zebrafish models for the nervous system have been established quite recently, as we have seen. However, for the visual system, zebrafish has been an important model for a long time now. One of the pioneers was Kljavin, who 36 years ago published a beautiful classical EM study using TEM and SEM (Kljavin, 1987). They described the early development of the retina in detail but focused more on the photoreceptors and outer segments. A few years later, Schmitt and Dowling built on this work and focused on the circuitry of the inner segments of the retina and took many TEM micrographs to describe the ultrastructure of the eye through development between 1.5 and 5 dpf (Schmitt & Dowling, 1999). Thus, these classical EM studies laid the groundwork for our understanding of zebrafish retina development and function.

As in hair cells, neurons in the retina possess ribbon synapses. The development of the cone photoreceptor ribbon synapses has first been described in detail by Biehlmaier and colleagues with TEM (Biehlmaier et al., 2003b). In a mutant screen, Allwardt and colleagues found fish that seemed to be completely blind. They investigated the retinal ultrastructure with TEM and discovered that the ribbon synapses of the photoreceptor neurons (but not the bipolar cells) were disrupted. The ribbons were still present and had vesicles tethered to them, but they were detached from the plasma membrane and were “free‐floating” in the synaptic terminals (Allwardt et al., 2001). We now know that this was caused by a mutation in *synaptojanin1*, which encodes a key player in the synaptic cycle.

Another mutation, *gnn*, was discovered to cause cone photoreceptor dystrophy and degeneration of the retinal pigment epithelium (Biehlmaier et al., 2003a). Via histology, immunohistochemistry, and TEM the authors described this mutant phenotype in detail and concluded that the early phases of developmental morphology of *gnn* mutants show similarities with age‐related macular degeneration in humans and the latter stages resemble degenerations seen in retinitis pigmentosa. The authors suggested that this mutant could be used as a model to study these diseases.

There is also a zebrafish model available for glaucoma, that is, degeneration of the optic nerve. Veth and colleagues used TEM and other methods to describe a mutant that causes myopia and other risk factors for glaucoma (Veth et al., 2011).

One of the causes of Usher syndrome type 1 is mutation of *myosin VIIAa*. Hodel and colleagues found that Myosin VIIAa is a specific marker for the cone accessory outer segment (AOS) in zebrafish (Hodel et al., 2014). The AOS runs alongside the photoreceptor outer segment and its function is still unknown. The authors suggest that future studies of the outer segment should pay more attention to the often‐neglected AOS. Their study is a nice example of the power of classical EM methods. They show the ultrastructure of the AOS with TEM, give an idea of the 3D appearance and location of the AOR via some impressive SEM of the retina, and utilize immunogold labeling to show the abundance of Myosin VIIAa in the AOR.

Classical EM methods are evidently just as useful as ever. And nowadays they are complemented by advanced, modern EM techniques. For example, Vishwanathan and colleagues used an advanced CLEM approach: They combined serial volume EM with two‐photon light microscopy calcium imaging to investigate a neural integrator of the oculomotor system (Vishwanathan et al., 2017). They identified neurons involved in eye movement by correlating their activity patterns (via calcium imaging) to the eye movements of zebrafish larvae. Then, they used an automatic tape‐collecting ultramicrotome (ATUM) to section a gigantic volume of 220 × 112 × 57 μm^3^ onto tape and imaged it via SEM. By correlating the electron micrographs to the calcium imaging data, they were able to investigate the ultrastructure and connectivity of the recorded neurons. Thus, they provided the first comprehensive morphological description of this neural integrator and offered evidence of synapses between integrator neurons for the first time.

## 7 CIRCUITS AND CONNECTOMICS

One important aspect for understanding nervous system function is circuitry, the synaptic connections. A map of the totality of the connections of a nervous system is called a connectome. To date, EM is the only method that can reliably reveal synaptic connections. Thus, the field of connectomics is entirely reliant on volumetric EM techniques. A beautiful example of this is a study by Wanner and colleagues, where they reconstructed the entire olfactory bulb of the zebrafish larva, including the projections, with SBEM (Wanner et al., 2016). To name just one of their interesting findings: It turns out that the organization of the olfactory bulb of the larva seems to be more similar to the insect antennal lobe than to the olfactory bulb of the adult fish. Later, Wanner and Friedrich reconstructed the full wiring diagram of this data set and discovered computational principles in the olfactory bulb (Wanner & Friedrich, 2020).

Just 1 year after the initial acquisition of the olfactory bulb, Hildebrand and colleagues published their data set of an *entire brain* of a 5.5 dpf larva in 18,000 sections (Hildebrand et al., 2017). Their heroic effort was made possible by the ATUM technique and by efficient targeted imaging at different scales, depending on resolution needs for reconstruction. To top it off, they also co‐registered functional reference atlases and in vivo two‐photon imaging data they had collected from the same specimen, setting a new benchmark for volumetric EM data sets. They made all their data available as a resource. Of note, they did not yet image at a resolution high enough to map synapses. In addition, cellular reconstructions are limited so far. However, with the ATUM technique, re‐imaging at higher resolutions is possible.

In another study using SBEM, the authors reconstructed the circuitry of two segments of the larval (6 dpf) spinal cord (Svara et al., 2018). They imaged a volume of 74 × 74 × 207 μm^3^, at a voxel size of 9 × 9 × 21 nm^3^. This means they had to image almost 10,000 sections of the spinal cord. To manage the tracing of this enormous data set, they had to rely on an army of trained students and external contractors. Thanks to their effort, they could show that one type of interneuron connects to specific motoneurons. This helps to understand how the larva manages to coordinate fast muscle contractions during escape responses.

Connectomes are undoubtedly crucial for understanding nervous systems, but they generally represent just snapshots of circuit architecture. Structure needs to be imbued with function. Luckily, with the small, transparent brains of zebrafish larvae, it is possible to obtain activity data from a whole brain region via multiphoton calcium imaging. Wanner and Vishwanathan published a very useful introduction and guide how to map synaptic connectivity to neuronal activity in zebrafish larvae (Wanner & Vishwanathan, 2018). As already mentioned above, they had used this technique to great effect in the olfactory bulb (Wanner et al., 2016) and also in the hindbrain (Vishwanathan et al., 2017).

In a more recent study, Vishwanathan, Yang, and colleagues analyzed the connectome of the larval brain stem (Vishwanathan et al., 2022; Yang et al., 2023). Remarkably, they were able to make functional predictions based on neural network modeling derived from the connectome. This result further underlines the promise of connectomics for predicting behavior.

One “problem” of connectomics data sets is the sheer amount of information. It can be difficult and extremely time consuming to analyze the data, even when focusing on a specific question. Choi and colleagues took the data set of Hildebrand and colleagues and developed a visual analytics system to facilitate its exploration (Choi et al., 2021). They called this tool “ZeVis.” ZeVis can combine 2D and 3D views of the EM data and overlay them with segmentations. This helps with analyzing and interpreting brain structures. A graph‐based interface facilitates customization of what is displayed. Their tool seems very helpful, and they used it to analyze the distributions of nuclei in several brain regions as an application example.

The latest advancement in large‐scale reconstruction of the zebrafish brain was just published last year by Svara and colleagues (Svara et al., 2022). They also acquired a whole brain of a larva (5 dpf) but instead of ATUM used SBEM to generate almost 29,000 sections with a constant voxel size of 14 × 14 × 25 nm^3^. Their work marks the first whole‐brain data set of a vertebrate with synaptic resolution. Another innovation of their work is that they developed a tool for automatic reconstruction of neuronal circuits on the synapse level, which is immediately accessible to the research community. Automation is the only way to go forward for dealing with the flood of data these volumetric EM techniques are giving us.

For an excellent recent review of connectomics in zebrafish, see Friedrich & Wanner (2021).

For an overview of the studies cited in this article, see Table 3. The tissues or diseases studied and the EM techniques used are also given for quick reference.

**TABLE 3:** Overview of the cited studies using electron microscopy techniques on the zebrafish nervous system.

Tissue or disease studied	Electron microscopy technique(s) used	Publication(s)
Cell culture	TEM	Ghosh et al., 1997
Brain explants	TEM, SEM	Peguero et al., 2022
Brain injury	STEM	Kuipers et al., 2016
Mercury poisoning	TEM	Cambier et al., 2012
Adult forebrain	TEM, SEM	Lindsey et al., 2012
Spinal neurons in early embryo	TEM	Kuwada et al., 1990
Spinal cord	SBEM	Svara et al., 2018
Neuromast	FIB‐SEM	Schieber et al., 2017
Myelin	TEM	Brösamle & Halpern, 2002 Pogoda et al., 2006
Myelin comparison between zebrafish and mouse	TEM, immunogold labeling	Siems et al., 2021
Mauthner neuron	TEM, array tomography (CLEM), SBEM	Kimmel et al., 1981 Strobel et al., 2020 Svara et al., 2022
Blood–brain barrier	TEM	Eliceiri et al., 2011 Azhari et al., 2021
Chemical synapses (larva)	ET	Helmprobst et al., 2015 Kaltdorf et al., 2017 Kaltdorf et al., 2018
Chemical synapses (adult)	TEM	Bayés et al., 2017
Alzheimer's disease	TEM	Bhattarai et al., 2016 Song & Pimplikar, 2012 Newman et al., 2017 Javed et al., 2019 Chen et al., 2020 Gao et al., 2022
Parkinson's disease	TEM	Flinn et al., 2009
Viral infection	TEM	Wang et al., 2021 Wang et al., 2022
Enteric nervous system	TEM	Baker et al., 2019
Epithelial paraneurons	TEM	König et al., 2019
Hair cells	SEM, TEM, SBEM	Harris et al., 2003 Obholzer et al., 2008 Riley et al., 2023 Dow et al., 2018
Retina	TEM	Kljavin, 1987 Schmitt & Dowling, 1999 Biehlmaier et al., 2003a Biehlmaier et al., 2003b Allwardt et al., 2001
Glaucoma	TEM	Veth et al., 2011
Usher syndrome type 1	TEM	Hodel et al., 2014
Larval oculomotor system (hindbrain)	SEM (ATUM), correlated with two‐photon calcium imaging	Vishwanathan et al., 2017
Complete larval olfactory bulb	SBEM	Wanner et al., 2016 Wanner & Friedrich, 2020
Larval brain stem	SEM (ATUM), correlated with two‐photon calcium imaging	Vishwanathan et al., 2022 Yang et al., 2023
Whole brain, 5.5 dpf	SEM (ATUM)	Hildebrand et al., 2017
Whole brain, 5 dpf	SBEM	Svara et al., 2022

## 8 CONCLUSION

As we have seen, zebrafish is a powerful model and EM is a powerful technique for studying the nervous system in health and disease. In fact, zebrafish is especially compatible with EM in many cases. The larvae can easily be fixed, embedded, and imaged, and their small size makes all these steps easier. Plus, they are one of the few vertebrate models that can be preserved via high‐pressure freezing as intact animals. Mouse embryos are not only much harder to access and larger, but they are also not yet free behaving. Zebrafish larvae already start hunting their own food at just 5 dpf and thus their nervous systems are functionally more developed than those of unborn mice.

These advantages make zebrafish into a model that can often compete with or complement mammalian models and may even be the better choice for many questions. It should be mentioned that the same can be true for other fish models, especially medaka (*Oryzias latipes*), which is also very well established as a model in biomedical research and has been used to study the nervous system with EM for decades. Practically all EM approaches performed in zebrafish should also be suitable for medaka, in principle.

As for EM as an approach, this review reinforces how it can be useful in many different models and projects. For revealing the ultrastructure, there is no alternative to EM and volumetric approaches enable connectomics on large scales. In the near future, acquisition of connectome data sets in zebrafish larvae will become a routine task. Thus, the entire nervous systems of mutants and disease models will be available. If automated data analysis approaches can keep up with this development, this could mark the next breakthrough in biomedical research of the nervous system.

## AUTHOR CONTRIBUTIONS

Sebastian M. Markert wrote the review.
